# Modification of Recycled Concrete Aggregate and Its Use in Concrete: An Overview of Research Progress

**DOI:** 10.3390/ma16227144

**Published:** 2023-11-13

**Authors:** Yingqiang Su, Yuchong Yao, Yang Wang, Xuan Zhao, Li Li, Jie Zhang

**Affiliations:** 1 Architectural Engineering Institute, Huzhou Vocational & Technical College, Huzhou 313002, China; 2022016@huvtc.edu.cn (Y.Y.); 2021004@huvtc.edu.cn (Y.W.); 2022027@huvtc.edu.cn (X.Z.); 2 Huzhou Key Laboratory of Green Building Technology, Huzhou 313002, China; 3 College of Water Resources and Architectural Engineering, Northwest A&F University, Yangling, Xianyang 712100, China; drlili@nwafu.edu.cn; 4 Department of Architectural Engineering, Jiyuan Vocational & Technical College, Jiyuan 459099, China; hylzj6@126.com

**Keywords:** waste concrete, recycled aggregate, adhesion mortar, modification technique

## Abstract

The differences in physical properties, chemical properties, and mechanical properties between reclaimed concrete aggregate and natural aggregate are discussed in this paper. In this paper, the commonly used improvement techniques of recycled concrete aggregate are reviewed. Physical modification involves peeling the attached mortar layer using mechanical and thermodynamic means, including mechanical grinding and shaping, heat treatment, and microwave or electric pulse treatment. Chemical modification is based on the chemical reaction of some materials with recycled aggregate attached mortar, including acid treatment removal, water glass strengthening, carbonation strengthening, inorganic slurry strengthening, and polymer strengthening. Microbial modification is mainly based on the metabolic activity of specific microorganisms that induce carbon deposition modification. The results show that the reinforced technology of recycled aggregate has made some progress in improving the performance of recycled aggregate, but there are still some problems, such as inconsistent strengthening effects and the unstable compatibility of composite materials. In this paper, future research directions, such as the development of new strengthening materials and the integration of multi-functional strengthening technology, are described in order to provide some theoretical support for the utilization of recycled concrete aggregate.

## 1. Introduction

Concrete is the most widely used material in construction projects. However, due to the acceleration of urbanization and the rapid development of construction projects, a substantial amount of abandoned concrete has resulted in severe environmental issues. Globally, approximately 10 Bt of construction and demolition waste (CDW) is generated each year [1,2,3,4,5]. Traditionally, waste concrete has often been treated as refuse, transported to landfills, or dumped in the natural environment, which not only leads to wasting of resources but also causes pollution and environmental degradation [6,7,8]. The concrete industry has a strong demand for aggregates, such as sand and stone, and the consumption and exploitation of natural sand and stone causes damage to the ecological environment. If construction waste, such as waste concrete, can be transformed into high-quality recycled aggregate, it can completely or partially replace natural sand and stone, which will effectively realize the recycling and reuse of resources [9]. Numerous investigators have studied the recycled aggregates (RAs)instead of natural aggregates in concrete [10,11,12,13] to realize the resource utilization of CDW, reduce the over-exploitation of natural aggregates, and maximize the ecological and environmental effects and economic benefits in the construction sector [14,15].

In recent years, the utilization of recycled concrete aggregates from waste concrete has garnered significant attention as a solution to this problem [16,17,18,19]. Recycled concrete aggregates are obtained through processes, such as crushing, screening, and washing of waste concrete, and exhibit physical and mechanical properties similar to natural aggregates [20,21,22,23,24]. The utilization of recycled concrete aggregates not only reduces the consumption of natural resources but also lowers the processing costs associated with waste concrete, thereby contributing to the achievement of sustainability goals [25,26,27,28,29]. The potential for the recycling and reuse of CDW is high [30]. There is a huge market interest to reuse and recycle aggregates derived from CDW in construction materials and projects [31,32,33,34,35,36,37,38,39,40,41,42]. A.M. Sagheer studied the feasibility of utilizing locally produced coarse recycled aggregate (RA) from demolition waste in the UAE for structural applications. Comprehensive reviews are presented in [43,44,45,46].

However, due to the uncertainty regarding the properties and quality of waste concrete, there are some challenges associated with the use of recycled aggregates in certain engineering applications [47]. For instance, recycled aggregates often exhibit a high porosity and increased water absorption [21], which can lead to reduced workability of concrete. Additionally, harmful substances may be present in recycled aggregates, adversely affecting the mechanical properties and durability of concrete [48,49]. Therefore, the modification of recycled aggregates to enhance their performance and adaptability has become a focal point and a significant challenge in current research [50]. The research results of D. Foti [51,52,53,54] in the fields of unconventional organic calcium aggregate, chemical depolymerized PET waste and straw fiber modified mortar can be used for reference in the improved design of waste concrete aggregate. A. Ferraro et al. studied the synthetic aggregate method using the incineration of fly ash and cement-slag from municipal solid waste [55,56,57].

This article aims to provide a comprehensive review of modification methods for recycled aggregates from waste concrete and explore how to improve the performance of recycled aggregate and its application effect in concrete by introducing appropriate modifiers or processes. Specifically, this article will focus on the following aspects:

1. Provide an overview of the physical and chemical properties of recycled concrete aggregates obtained from waste concrete.
2. Review existing methods and technologies for modifying recycled aggregates, including physical, chemical, and biological approaches.
3. Summarize the current state of modification techniques, evaluate their pros and cons, and propose future research directions.

Through the research presented in this article, it is anticipated that new ideas and methods for the modification of recycled aggregates from waste concrete can be provided. This, in turn, will facilitate the widespread utilization of recycled aggregates in engineering practice and promote the direction of sustainable development in construction projects.

## 2. Properties of Recycled Concrete Aggregates

Recycled concrete aggregates from waste concrete and natural aggregates are two distinct materials with several significant differences in terms of physical properties, chemical properties, mechanical performance, and sustainability [58,59]. The following sections will provide a detailed explanation of these differences and explore their impacts on concrete properties and engineering applications.

### 2.1. Physical Properties

**Figure 1:**
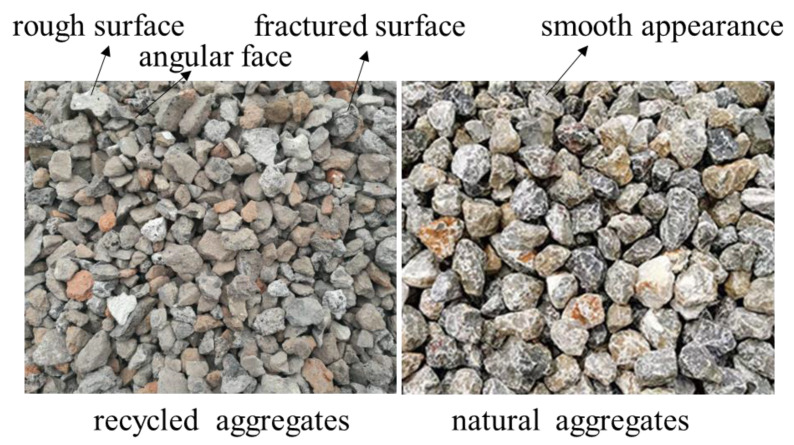
Comparison of particle shapes of recycled aggregates and natural aggregates.

**Figure 2:**
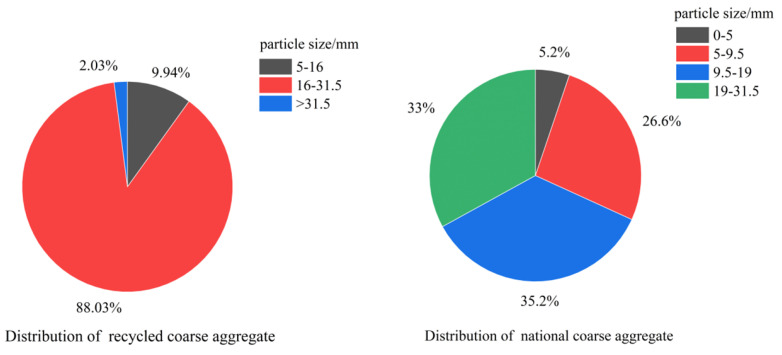
Comparison of the distribution ratio of each particle size of recycled coarse aggregate and natural coarse aggregate.

Particle Shape and Gradation: Recycled concrete aggregates from waste concrete typically have a more irregular particle shape compared to natural aggregates. The particle shape of recycled aggregates can be fractured or angular or have rough surfaces, whereas natural aggregates tend to have smoother and more uniform particle shapes. There are noticeable differences in the particle size distribution (gradation) between recycled coarse aggregates and natural coarse aggregates, leading to poor gradation [60]. Santamarina and Cho [61] summarized particle shape irregularity into three main scales: sphericity, roundness, and smoothness. A comparison of particle shape of recycled aggregates and natural aggregates is shown in Figure 1. J. Xiao [60] gave the particle size ratios of recycled coarse aggregate with a particle size of 16~31.5 mm and natural coarse aggregate with a particle size of 5~31.5 mm, as shown in Figure 2. It can be seen that the ratio of each particle size of recycled coarse aggregate is different from that of natural coarse aggregate.

Surface Characteristics: Due to the presence of a significant amount of adhering mortar on the surface of recycled aggregates, they are usually rougher in texture and contain more fine particles, capillary pores, and cracks resulting from the crushing process, whereas the surface of natural aggregates is relatively smoother [26].

**Figure 3:**
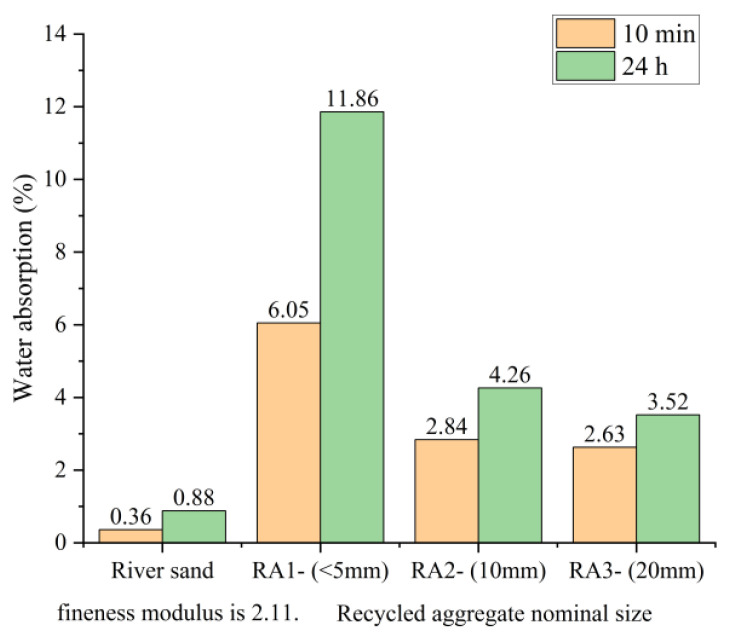
The water absorption rates of river sand and recycled aggregates with different particle sizes.

**Figure 4:**
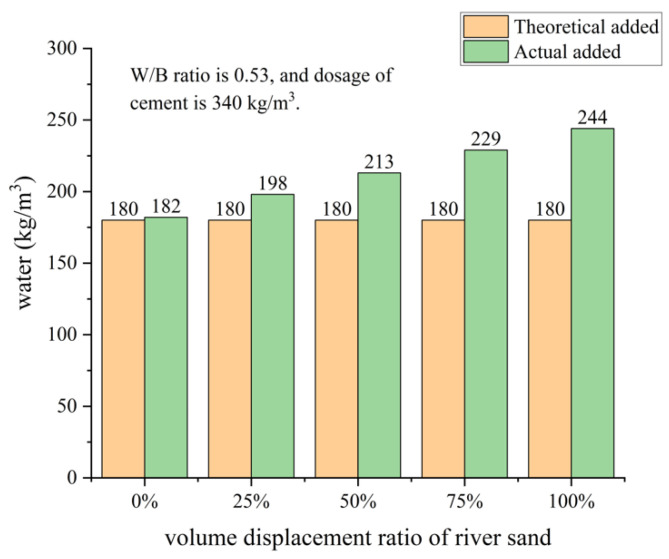
The effects of the amount of water used in the mixes on different volume displacement ratios of river sand.

Water Absorption: The differences in surface characteristics between the two result in higher water absorption for recycled aggregates compared to natural aggregates, making recycled aggregates more prone to absorbing and retaining moisture. This can lead to reduced workability of concrete and an increase in the water–cement ratio. S.C. Kou [57] experimentally found that the water absorption of recycled aggregates of different particle sizes was higher than that of river sand. In addition, the smaller the particle size of recycled aggregates, the higher the water absorption. The water absorption rates of river sand and recycled aggregates of different particle sizes are shown in Figure 3. The theoretical water consumption in the mixes are based on all the aggregates being in a saturated surface dry condition. The amount of water added to the mixes was higher due to the high water absorption of the recycled aggregates. The effect of the amount of water used in the mixes on different volume displacement ratios of river sand is shown in Figure 4.

### 2.2. Chemical Properties

Cement Hydration Products: Recycled concrete aggregates from waste concrete carry a significant amount of cementitious mortar on their surfaces, and as a result, they contain a substantial amount of cement hydration products, such as C-S-H, calcium hydroxide, ettringite, etc., with an interface transition zone typically around 40–50 μm in width [62]. In contrast, natural aggregates primarily consist of components, like calcium carbonate and silica dioxide. Therefore, there are substantial differences in the chemical composition and properties between recycled concrete aggregates from waste concrete and natural aggregates. The surface composition of CaCO_3_ particles has a stronger adsorption capacity for ions (mainly Ca^2+^) in the slurry. However, the adsorption capacity of particles with silicate phase on the surface is weak. A relatively strong bond (possibly an ionic-covalent bond) was formed between CaCO_3_ and C-S-H through the chemical adsorption of calcium ions in the pore solution of cement slurry by limestone, which made the nucleation and growth of C-S-H on CaCO_3_ surface faster and the growth of C-S-H directional [63]. However, due to the uneven surface of recycled concrete aggregate and the presence of a large amount of calcium hydroxide, hydration products are difficult to nucleate and grow steadily on its surface, so more calcium hydroxide will be generated [64].

Harmful Substances: Recycled concrete aggregates from waste concrete, especially those obtained from coastal or saline-alkali environments, may contain harmful substances, such as chloride ions, sulphates, organic materials, and other construction debris. These harmful substances can have adverse effects on the mechanical properties, durability, and resistance to chemical corrosion of concrete. In contrast, qualified natural aggregates typically do not contain harmful substances. When sulfuric acid is mixed into regenerated aggregate, the expansion failure of concrete is easy to occur, shown as Equation (1). When chlorine ions are mixed, the steel bar is prone to electrochemical corrosion, resulting in corrosion of the steel bar, shown as Equation (2).

C_4_AH_18_ + 2CH + 3SO_4_^2−^ + 12H = C_6_AŜ_3_H_32_ (1)

Fe^2+^ + 2OH^−^= Fe(OH)_2_ (2)

### 2.3. Mechanical Performance

Strength: Recycled concrete aggregates from waste concrete typically exhibit lower strength compared to natural aggregates. The particles in recycled aggregates may have microcracks or fractured surfaces, and their gradation is often poorer, all of which contribute to their lower crushing values. W. Li [65] experimentally demonstrated that the compressive strength of recycled concrete increased first and then decreased with the increase in recycled aggregate content according to the optimal gradation. When the content of recycled coarse aggregate is 30%, 50%, 70%, and 100%, the 28 d compressive strength of recycled concrete increases by 14%, 7.9%, −15.9%, and −18.6%, respectively, compared with ordinary concrete (the minus sign means decrease). The tensile strength of recycled concrete decreases gradually with the increase in recycled aggregate content.

Interparticle Bonding: Due to their particle shape and the presence of mortar layers on their surfaces, recycled aggregates have weaker interparticle bonding with the cementitious matrix. This can result in reduced cohesion and crack resistance of the concrete.

### 2.4. Sustainability

Resource Utilization: The utilization of recycled concrete aggregates from waste concrete can reduce the consumption of natural resources and mitigate the environmental damage caused by mining activities. In contrast, natural aggregates need to be obtained through mining and other related processing.

Environmental Impact: The use of recycled aggregates can reduce the landfilling and dumping of waste concrete, minimizing environmental pollution and the wastage of land resources. Furthermore, the production process of recycled aggregates is typically more energy efficient and emission reducing compared to the production process of natural aggregates, significantly reducing the carbon emissions associated with concrete. T. Zhang et al. [66] mentioned that the use of carbonized solid waste in mortar and concrete can significantly reduce CO_2_ emissions, and 2035.4 Mt CO_2_ emissions can be reduced in one year in the Asia-Pacific region alone. The data of typical countries are shown in Table 1.

### 2.5. Impact on Concrete Performance and Engineering Applications

The presence of multiple interface transition zones in recycled aggregates [62], coupled with their lower strength, can lead to a reduction in concrete’s strength and bonding with reinforcing steel. For example, the compressive strength of concrete decreases as the replacement rate of recycled aggregates increases, with a compressive strength of only 55% for regular concrete when 100% of the fine aggregates are replaced with recycled aggregates [67]. Parameters, such as electrical conductivity and the chloride ion diffusion coefficient in concrete, exhibit linear increases with higher rates of recycled aggregate substitution [68]. Bao et al. [26] found that the inclusion of recycled coarse aggregates generally lowered the compressive strength of concrete and increased the transport coefficients of water and chloride ions. Therefore, optimizing mix designs, employing admixtures, and utilizing aggregate modification techniques are necessary to enhance concrete performance. K. Pandurangan et al. studied the factors affecting the bond strength of recycled aggregate concrete using different treatment methods [69,70,71].

**Figure 5:**
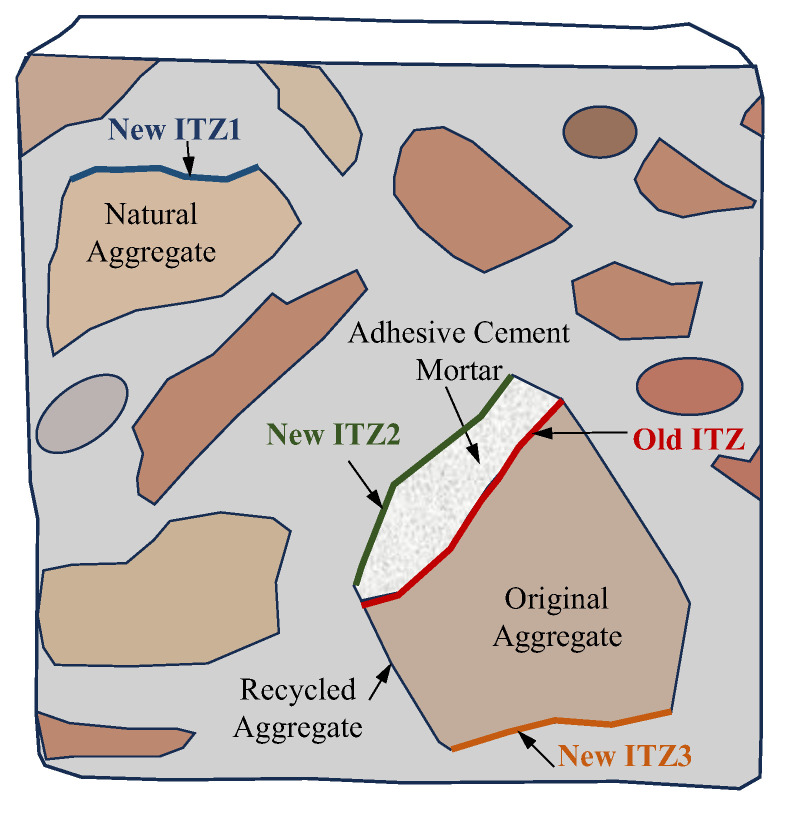
Schematic diagram of the interface structure in recycled aggregate concrete [72].

The interface structure diagram of recycled aggregate concrete [72] is shown in Figure 5. Here, the old ITZ is the old interface transition zone between the original natural aggregate in the recycled aggregate and the hardened porous adhesive cement mortar, and the new ITZ1 is the new interface transition zone between the ordinary natural aggregate and the newly mixed cement mortar. ITZ2 is a new interfacial transition zone between the porous adhesive cement mortar hardened by recycled aggregate and the newly mixed cement mortar, and a new interfacial transition zone is formed between the original natural aggregate and the newly mixed cement mortar in the new ITZ3 recycled aggregate.

In summary, recycled concrete aggregates from waste concrete and natural aggregates exhibit several significant differences in terms of physical properties, chemical properties, mechanical performance, and sustainability. Understanding these differences is of paramount importance for the enhancement of recycled aggregate modification techniques, the judicious utilization of recycled aggregates, the optimization of concrete mix designs, and the improvement of engineering applications.

## 3. Recycled Concrete Aggregate Modification Techniques

### 3.1. Physical Modification

Physical enhancement involves applying stress to the adhering mortar layer through mechanical and thermomechanical approaches to detach it, thereby improving the performance of recycled aggregates. Physical enhancement techniques can be categorized into three main types: mechanical grinding and shaping, heat treatment, and electric pulse treatment.

#### 3.1.1. Mechanical Grinding and Shaping

**Figure 6:**
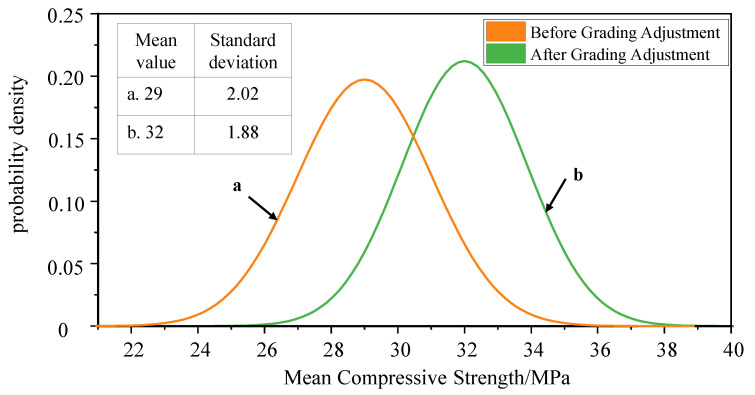
Normal distribution curve of compressive strength with a replacement ratio of 60% before and after gradation modification [60].

By subjecting waste concrete to mechanical grinding and sieving, the physical properties and particle shape of recycled aggregates can be improved, enhancing their performance in concrete applications. This includes the following:

1. Crushing and Sieving: This is the most fundamental and commonly used method for preparing and processing recycled aggregates. Waste concrete is crushed using crushing equipment, and then the crushed concrete is graded and reassembled through sieving equipment to obtain recycled aggregates that meet grading and physical performance requirements. For example, Wang et al. [73] found that the regenerated aggregate was prepared according to the aggregate size of 10–20 mm and 5–10 mm at a mass ratio of 2:1 to obtain continuously graded aggregate, numbered RA1. The 5–10 mm granular aggregate is screened through the 4.75 mm screen to remove the part less than 4.75 mm. After passing through the sieve, the 10–20 mm granular aggregate and the 5–10 mm granular aggregate are blended to obtain continuous graded aggregate, which is termed number RA2. The 24 h water absorption of RA1 and RA2 was 4.99% and 4.78%, respectively. The results indicated that the water absorption of regenerated aggregate decreased significantly after the removal of small particle size aggregate and fine powder below 4.75 mm by sieving. Xiao et al. [60] noted that adjusting the aggregate grading resulted in a significant reduction in the standard deviation of compressive strength for concrete, with an increase in the mean value. The histogram and curve of the normal distribution of compressive strength when the substitution rate is 60% before and after allocation are shown in Figure 6. It can be seen from the figure that manual gradation adjustment can increase the mean value of the normal distribution curve and reduce the standard deviation of the normal distribution curve, thus increasing the representative value of compressive strength. The compressive strength of recycled concrete is improved, and the dispersion of performance is effectively controlled.
2. Air Separation: Air separation is based on the differences in particle size and density within recycled aggregates and relies on the action of wind to segregate particles. Heavier particles are less easily carried by the wind due to inertia, while lighter particles are more prone to being carried away by the wind, achieving particle separation. For example, Li Gen [74] subjected crushed waste concrete particles with a particle size smaller than 4.75 mm to 15 min of vibration grinding followed by air separation at a velocity of 16 m/s to obtain recycled fine aggregates that meet the required specifications.
3. Vibration Mixing Pretreatment: Preprocessing recycled aggregates through abrasion and crushing within a concrete mixer can conveniently achieve mechanical grinding and shaping of recycled aggregates. Wang Bo [72] conducted experiments using two types of mixers (double-shaft mixer and planetary mixer), two mixing speeds (30 r/min and 55 r/min), and two mixing methods (vibration and regular) to mix crushed waste concrete recycled aggregates. The results show that the fracture of reclaimed aggregate is the most obvious in the double horizontal shaft mixer, followed by the vibration mixing, and the planetary mixing is the least obvious in the planetary mixer. The wear phenomenon is the most obvious in vibration stirring mode, followed by ordinary forced stirring mode, and the least wear is noted with planetary stirring. The larger the stirring speed, the longer the stirring time, and the more obvious the abrasion breaking phenomenon. As the stirring time increases, the particles that have been worn and broken will have a secondary abrasion breaking phenomenon. The wear particles can decrease the slump of recycled concrete and affect the working performance. The wear particles can improve the compressive strength of concrete only under the vibration mixing mode, while the broken particles can increase the compressive strength and slump of concrete.

These mechanical grinding and shaping methods can improve the physical properties and particle shape of recycled concrete aggregates from waste concrete, making them more suitable for use in concrete. However, it is notable that the mechanical grinding and shaping process may exacerbate surface damage to recycled aggregates, leading to some performance degradation, such as reduced strength and increased water absorption [72].

#### 3.1.2. Heat Treatment

**Figure 7:**
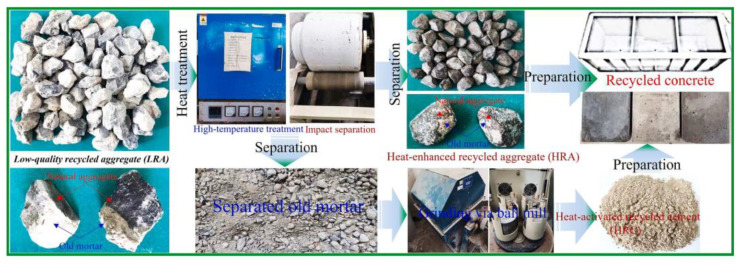
Making LRA into HRA and HRC using heat treatment [24].

Recycled concrete aggregates from waste concrete are subjected to high-temperature environments, involving hot air, hot water, steam, or thermal treatment. The application of high temperatures serves to remove cementitious gel and other organic substances from the surface of recycled aggregates, enhancing the cleanliness of their particle surfaces. Simultaneously, high-temperature treatment can improve the particle shape of recycled aggregates, making them more uniform and regular. A typical process flow for preparing concrete using heat-treated recycled aggregates is illustrated in Figure 7.

However, due to the variability in raw materials for recycled aggregates, differences in particle size, and moisture content, there is no definitive consensus in the literature regarding the optimal heat treatment temperature and regimen, leading to several contradictions. For instance, Li Gen [74] found that an ascent rate of 10 K/min and a heating temperature of 500 °C were the most significant conditions for disrupting the interface transition zone on the surface of recycled coarse aggregates, representing the optimal conditions for removing adhering mortar. On the other hand, Wu et al. [20] discovered that using a heat treatment of 700 °C could transform low-quality recycled aggregates into thermally enhanced recycled aggregates (HRA) as a substitute for natural aggregates and thermally activated recycled cement (HRC) as a substitute for ordinary Portland cement. Furthermore, the high energy consumption associated with heat treatment limits its widespread application.

#### 3.1.3. Microwave or Electric Pulse Treatment

**Figure 8:**
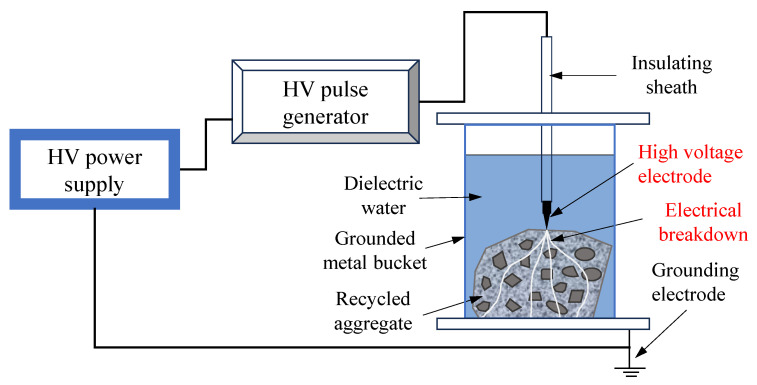
Scheme of the device used for electrohydraulic fragmentation experiments [82].

The modification method involving microwave or electric pulse treatment of recycled concrete aggregates from waste concrete is based on the application of electric pulse or microwave technology. By applying high-voltage electric pulses or microwaves to recycled aggregates, strong electric fields and current effects can be generated, leading to ion migration, electrochemical reactions, and electrothermal effects. These effects can alter the physical structure, surface properties, and chemical composition of recycled aggregates, thereby achieving the modification effect. The typical process for electric pulse treatment of recycled aggregates is illustrated in Figure 8.

Xiao Jianzhuang et al. [75] conducted a strengthening test on recycled coarse aggregate (RCA) using a low-power microwave, and compared with the traditional technology, microwave heating of modified recycled coarse aggregate had a better effect. Tsujino et al. [76] first proposed the new microwave technology for concrete aggregate recovery. CO_2_ emission testing and mechanical property testing during the treatment of 1 t waste concrete confirmed that the CO_2_ content generated in the microwave heating process was lower and that the recovered RCA quality was higher compared with traditional recycling methods. Ong et al. [77] systematically discussed the possibility of microwave technology in the recycling process of concrete and discussed the basic working principle of microwave technology in the application of concrete regeneration. Compared with other traditional aggregate extraction technologies, microwave heating combined with mechanical crushing technology can obtain higher RCA quality. It should be noted that due to the lack of relevant theoretical research, microwave technology has not been applied in the field of civil engineering on a large scale. On the one hand, concrete is a complex artificial composite material, and the properties of concrete under microwaves are affected by factors, such as the mix ratio and water content. On the other hand, the mechanisms of water migration, pore pressure, and thermal stress in concrete under high temperature are still unclear, which greatly limits the popularization and application of microwave technology [78].

Du Wenping [79] adopted an experimental research method and used microwave-assisted mechanical picking technology to treat waste concrete blocks, obtain high-quality recycled coarse aggregate, and then prepare recycled concrete to study its compressive properties and failure patterns. The mechanical properties of reclaimed coarse aggregate with high quality were compared with those obtained from natural coarse aggregate and other picking processes, which showed that the microwave-assisted mechanical picking process had strong advantages. The damage degree of regenerated coarse aggregate was analyzed from the microscopic point of view using a Scanning Electron Microscope (SEM) scanning test. The results showed that the coarse aggregate obtained by microwave-assisted mechanical process picking waste concrete blocks had high quality characteristics [80,81].

Menard et al. [82] found that both microwave and electric pulse technologies are effective in removing adhering mortar from the surface of recycled aggregates. Compared to microwave treatment, electric pulse technology has lower energy consumption (1~3 vs. 10~40 kW h t^−1^), but it can only be used for recycled aggregates immersed in water, posing some challenges for engineering applications. It is important to note that the electric pulse treatment modification method for recycled concrete aggregates from waste concrete is still in the research and development stage, and specific treatment parameters and process conditions need further optimization and validation.

### 3.2. Chemical Modification

#### 3.2.1. Acid Treatment for Adhering Mortar Removal

The acid treatment modification method for recycled concrete aggregates from waste concrete is based on the chemical reaction between acidic solutions and recycled aggregates. Commonly used acid wash solutions include hydrochloric acid, sulfuric acid, acetic acid, phosphoric acid, and others. The basic reaction equations are shown in Equations (3)–(5). These acidic solutions can chemically react with the adhering substances on the surface of recycled aggregates, such as cementitious materials, and pollutants, causing them to dissolve or detach, thus removing surface adherents. The acid treatment modification method for recycled concrete aggregates from waste concrete can effectively remove cementitious materials and pollutants, improve particle shape, increase the active sites and chemical reactivity on the surface of recycled aggregates, and enhance their bonding ability and interaction with the cementitious matrix.

(3) $$\mathrm{CaO}+2H^{+}\to {\mathrm{Ca}}^{2+}+H_{2}O$$

(4) $${\mathrm{Fe}}_{2}O_{3}+6H^{+}\to 2{\mathrm{Fe}}^{3+}+3H_{2}O$$

(5) $${\mathrm{Al}}_{2}O_{3}+6H^{+}\to 2{\mathrm{Al}}^{3+}+3H_{2}O$$

For example, Pan’s research [1] found that the water absorption of recycled aggregates soaked in 0.2 M acetic acid solution for 24 h decreased by up to 25.5%, and the apparent density and crushing index of all recycled aggregates treated with dilute acetic acid were improved. However, pickling treatment involves the treatment of acidic solutions and waste liquids, which can easily lead to safety and environmental issues, making it difficult to widely apply.

#### 3.2.2. Sodium Silicate Enhancement of Adhering Mortar

The method of sodium silicate enhancement for recycled aggregates is based on the chemical reaction and cementing action between sodium silicate and recycled aggregates. Sodium silicate is an inorganic binder material with excellent adhesion and cementitious properties. During the treatment process, the sodium silicate solution can penetrate the interior of the recycled aggregate particles, react chemically with the cementitious materials inside, form hydration products of the cementitious materials, and create a bond with the cementitious matrix, thus enhancing the recycled aggregates. The basic reaction equation is shown in Equation (6) [83].

(6) $$xH_{2}{O+\mathrm{Na}}_{2}O\cdot n{\mathrm{SiO}}_{2}+\mathrm{Ca}{\left(\mathrm{OH}\right)}_{2}\to \mathrm{CaO}\cdot n{\mathrm{SiO}}_{2}\cdot xH_{2}O+2\mathrm{NaOH}$$

Song et al. [84] found that after immersion in sodium silicate with a modulus of 2.8, the strength of recycled coarse aggregate increased, water absorption decreased, and the concrete strength increased by more than 50%, indicating a significant modification effect. Sodium silicate can also synergize with other nanomaterials to modify recycled aggregates. For example, Liu et al. [85] discovered that compared to control samples, the compressive strength and tensile strength of recycled aggregate concrete modified with a combination of sodium silicate and nano-silica increased by 25.3% and 21.1%, respectively, at 56 days. However, the introduction of sodium silicate, which is strongly alkaline, may lead to more severe alkali-aggregate reactions [83].

#### 3.2.3. Carbonation Reinforcement of Adhesive Mortar

**Figure 9:**
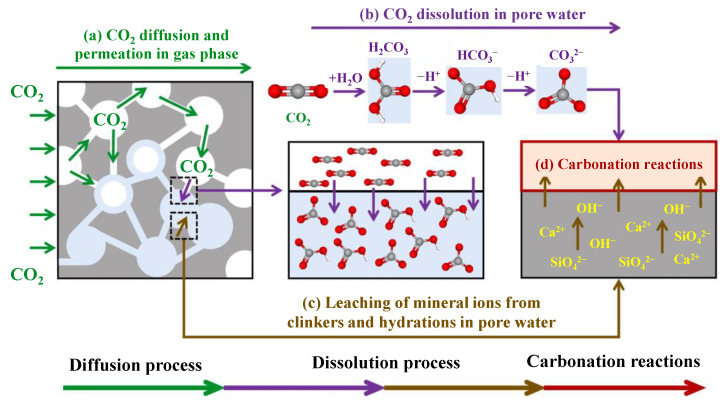
Illustration of physical and chemical carbonation processes of recycled concrete [66].

The carbonation reinforcement method for recycled aggregates is based on the chemical reaction and cementing action between carbonates and the adhesive mortar. In the process, recycled aggregates are typically exposed to carbon dioxide (CO_2_), which reacts with carbonates in the gas phase or solution to form a carbonate layer on the surface of recycled aggregates. The basic reaction equations for the oxidation of calcium, the hydration of calcium silicate gel, and the carbonation of ettringite are shown in Equations (7)–(9) [50]. Specifically, the carbonation process is influenced by the characteristics of the parent material of recycled aggregates, including particle size, porosity, moisture content, and the carbonation method and conditions, such as CO_2_ concentration, gas pressure, carbonation reaction time, relative humidity, and temperature. The carbonation mechanism of recycled aggregates can be described by the diffusion-dissolution-carbonation reaction process, as shown in Figure 9 [66]. The carbonate layer can fill the pores and microcracks on the surface of particles, improving the compactness and strength of recycled aggregates.

(7) $$\mathrm{Ca}{\left(\mathrm{OH}\right)}_{2}+{\mathrm{CO}}_{2}\to {\mathrm{CaCO}}_{3}+H_{2}O$$

(8) $$C-S-H\;\left(\mathrm{High}\;\mathrm{Calcium}-\mathrm{to}-\mathrm{Silicon}\;\mathrm{Ratio}\right){+\mathrm{CO}}_{2}\to C-S-H\;\left(\mathrm{Low}\;\mathrm{Calcium}-\mathrm{to}-\mathrm{Silicon}\;\mathrm{Ratio}\right)+{\mathrm{CaCO}}_{3}+H_{2}O$$

(9) $$C_{3}A\cdot 3{\mathrm{CaSO}}_{4}\cdot 32H_{2}O+{\mathrm{CO}}_{2}\to C_{3}A\cdot {\mathrm{CaSO}}_{4}\cdot 12H_{2}O+{\mathrm{CaCO}}_{3}+H_{2}O\;$$

Carbonation treatment can improve the shape and surface characteristics of recycled aggregate particles, increase the bonding force between particles, and enhance the compactness and mechanical properties of concrete. For example, after carbonation treatment, the loose bulk density of recycled coarse aggregates increased by 6.9%, and the compacted bulk density increased by 4.9% [73].

Carbonation strengthening can reduce the pores and microcracks in recycled aggregates, lowering the rate of water absorption and the impact of corrosive media. For instance, the water absorption rate of recycled coarse aggregates decreased by 2.1% after carbonation treatment for 24 h [73]. Experimental research by Song et al. [86] found that when the replacement rate of recycled coarse aggregates was 50%, 75%, and 100%, the chloride ion penetration resistance of carbonation-strengthened recycled aggregate concrete increased by 13.8%, 16.6%, and 11.3%, respectively.

It should be noted that carbonation strengthening of recycled aggregates requires control of conditions, such as CO_2_ concentration, pressure, addition of calcium hydroxide, and aggregate particle size, to ensure the appropriate carbonation reaction and strengthening effect.

#### 3.2.4. Strengthening Recycled Aggregates with Inorganic Slurries, such as Cement, Mineral Admixtures, and Nanomaterials

Basic Principle: The method of strengthening recycled aggregates with inorganic slurries, such as cement, mineral admixtures, and nanomaterials, is based on the cementitious reaction and encapsulation between them. During the treatment process, recycled aggregates usually come into contact with the slurry, and particles of cement, mineral admixtures, or nanomaterials react with the surface of recycled aggregates through hydration or pozzolanic reactions, forming a gel or encapsulation layer. Li Long et al. [82] adopted different modification methods for recycled coarse aggregate (RCA), that is, spraying colloidal nano-silica (NS) with different particle sizes and silica powder (SF) with a particle size of 420 nm on the surface of RCA to evaluate their efficiency and improve the performance of recycled aggregate concrete (RAC). The test results show that after spraying colloidal NS and SF on the surface of RCA, the mechanical properties and durability of RAC are significantly improved. In addition, the larger the particle size of NS, the more obvious the performance enhancement effect.

Cement slurry reinforcement treatment can create a dense cement gel layer or coating on the surface of recycled aggregates, filling the pores and micro-cracks on the particle surface, enhancing the compactness and strength of the recycled aggregates [87,88,89,90,91] and thus enhancing the compressive strength of concrete. Wang et al. [92] used the cement slurry wrapping method and secondary mixing process to strengthen recycled coarse aggregates, and the 28-day compressive strength of C20, C30, and C40 recycled concrete reached 98%, 91%, and 97% of that of ordinary concrete, respectively. After modifying recycled coarse aggregates with sprayed colloidal silica ash, the compressive strength and elastic modulus of the corresponding recycled concrete increased by 9.2% and 11.7%. Its durability even approached that of natural aggregate concrete. The secondary water absorption rate, charge transfer value, and carbonation depth decreased by 66.3%, 46.1%, and 28.4%, respectively [93].

Improved Particle Shape: Strengthening treatment can improve the shape and surface characteristics of recycled aggregate particles, increasing the bulk density. For example, after soaking in 2 wt.% and 4 wt.% colloidal silica solutions, the loose bulk density of recycled coarse aggregates increased by 15.6% and 18.6%, respectively [73].

Cement, mineral admixtures, and nanomaterial slurry reinforcement treatment can reduce the pores and micro-cracks in recycled aggregates, reduce the rate of water and chloride ion penetration, and improve the durability and impermeability of concrete. For instance, after soaking in 2 wt.% and 4 wt.% colloidal silica solutions, the water absorption rate of recycled coarse aggregates decreased by 3.65% and 4.67%, respectively [73].

It should be noted that the cement, mineral admixtures, and nanomaterial reinforcement treatment of recycled aggregates require control of the slurry’s proportions, treatment time, and environmental conditions to ensure appropriate curing reactions and reinforcement effects. Additionally, recycled aggregates treated with cement slurry reinforcement need to undergo proper screening and processing to achieve the required particle grading.

#### 3.2.5. Polymer Reinforcement of Recycled Aggregates

Polymer reinforcement of recycled aggregates is based on the interaction and encapsulation between polymer materials and recycled aggregates. During the treatment process, recycled aggregates typically come into contact with a polymer solution or molten polymer, and polymer materials interact with the surface of the recycled aggregates through adsorption, penetration, or encapsulation. This interaction can improve the surface properties of recycled aggregates and the bond between particles, enhancing their mechanical performance and stability.

**Figure 10:**
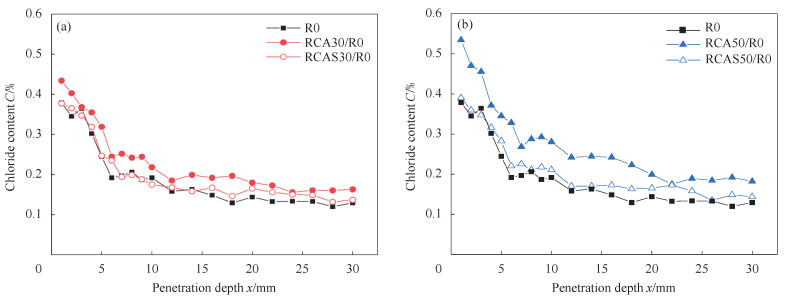
Chloride profiles of recycled aggregate concrete [49]. (**a**) Chloride profiles of RCA30/R0 recycled aggregate concrete with different aggregate types. (**b**) Chloride profiles of RCA50/R0 recycled aggregate concrete with different aggregate types.

Bao et al. [49] found that after silane impregnation treatment, the water absorption rate of recycled coarse aggregates was effectively controlled, resulting in a significant improvement in the chloride ion permeability of the prepared concrete. The internal chloride ion content of C30 and C50 recycled concrete was significantly reduced, approaching that of ordinary concrete, as shown in Figure 10. Li [94] found that water-based epoxy and styrene-butadiene latex-modified recycled coarse aggregates improved the compressive and flexural strength of pervious bricks and improved their permeability. Song et al. [84] found that a 20% concentration of polyaluminum sulphate had a strengthening effect on the strength and water absorption of recycled coarse aggregates from waste concrete. The concrete strength increased by approximately 30%, and durability also improved. However, the overall effect was slightly inferior to that of sodium silicate treatment. Additionally, some studies have found that polymer-treated recycled aggregates may lead to a slight reduction in concrete strength [49]. D. Foti [54] found that polyethylene terephthalate (PET) had an effect on the mix ratio of concrete.

### 3.3. Microbial-Induced Carbonate Precipitation Modification

**Figure 11:**
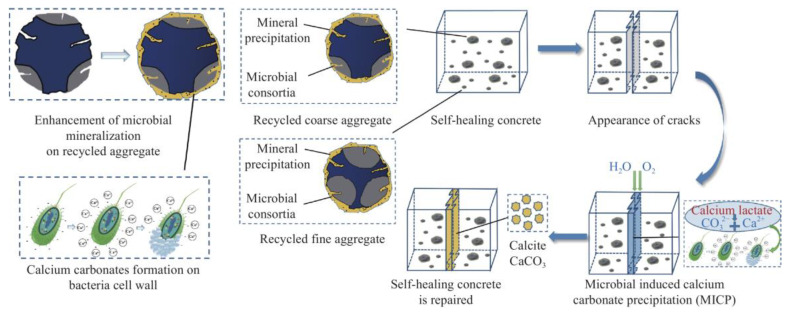
Crack self-healing mechanism of concrete incorporating bacteria into recycled aggregate (RA) with enhancement [95].

Microbial-induced carbonate precipitation (MICP) is a method used for enhancing recycled aggregates, based on the metabolic activity of specific microorganisms, with the most commonly used microorganisms being urease-producing bacteria (such as urease-producing Bacillus). During the treatment process, recycled aggregates make contact with a solution containing urea and a source of calcium. Microorganisms metabolize urea, breaking it down into ammonia and carbon dioxide. Ammonia reacts with calcium ions in the solution to generate calcium carbonate. Subsequently, calcium carbonate precipitates on the surface of the recycled aggregates, forming a calcium-rich cementitious material that fills the pores and microcracks on the particle surface, thereby enhancing their mechanical properties and stability. The principle of microbial mineralization in the repair of cracks in recycled aggregates is shown in Figure 11 [95]. MICP can improve the compressive strength, enhance particle shape, and increase durability. Moreover, it is environmentally friendly, with low energy consumption and minimal environmental impacts.

For instance, research conducted by Zhang et al. [96] indicates that Bacillus spores of cocci bacteria-induced calcium carbonate deposition have a favorable strengthening effect on recycled coarse aggregates. The crushing index and water absorption significantly decrease, while the apparent density increases. The optimal strengthening effect is achieved at 10 days. It is important to note that microbial-induced carbonate precipitation for enhancing recycled aggregates requires control over the microbial growth conditions, the concentrations of urea and calcium sources, and the treatment duration to ensure appropriate carbonate deposition and strengthening effects. Hua Sujin et al. [95] discovered that using a mixed culture of microorganisms for enhancing recycled aggregates can yield good enhancement results. After 28 days of repair, the average crack width in the concrete and the complete repair rate reach 0.28 millimeters and over 60%, respectively. This represents improvements of over 47.4% and 50%, respectively, compared to regular concrete without microbial-induced mineralization for crack repair. Furthermore, the water permeability coefficient of the repaired concrete decreases by over 99.7%, demonstrating better waterproofing performance.

**Figure 12:**
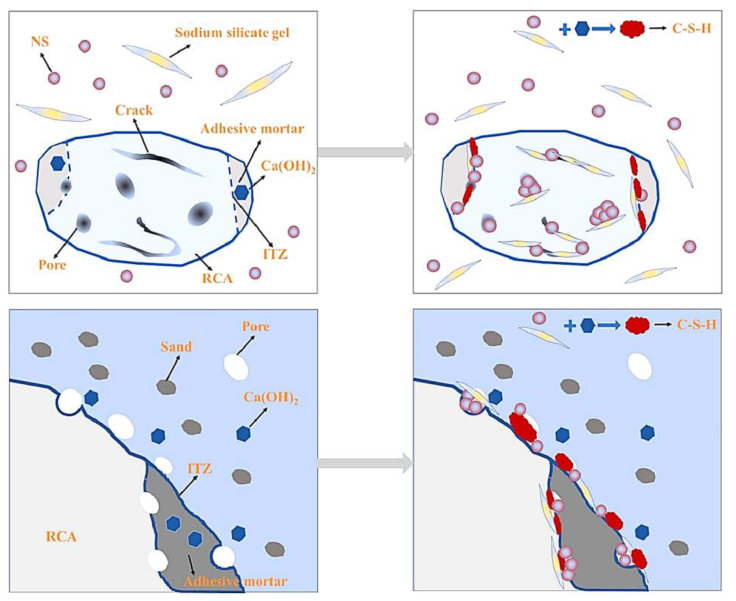
Schematic illustration of the NMS modification effect on the macro performance of MRAC [85].

It is notable that the combination of the above methods is often used to achieve better results. Therefore, flexibility in combining these methods is recommended for practical applications. For example, Xue [97] found that the combination of sodium silicate enhancement and cement-based penetrating crystalline material slurry pre-wetting is optimal for enhancing recycled coarse aggregates to prepare concrete with improved resistance to chloride ion penetration and freeze-thaw resistance. The principle of the sodium silicate and nano silica co-modification of recycled aggregates [85] is illustrated in Figure 12. Specifically, nano silica (NS) and sodium silicate can penetrate into the interior of the recycled coarse aggregates through microcracks and strengthen the microstructure of the old interfacial transition zone (ITZ). During the formation of the new ITZ, NS and sodium silicate can enter the interior of the recycled coarse aggregates through microcracks and react with calcium hydroxide, converting it into C-S-H gel and thereby enhancing the ITZ. Modified recycled aggregate concrete has a relatively higher degree of hydration, improved pore structure, and increased strength and durability of recycled concrete.

## 4. Conclusions and Outlook

The research shows that the production and recovery process of recycled aggregate is accompanied by high energy consumption, a lot of dust pollution, and other problems. In addition, the quality of recycled aggregate produced is not high, and it exhibits poor adaptability [98,99,100,101]. The existing physical, chemical, and biological techniques for enhancing recycled concrete aggregate (RCA) have made some progress in improving the performance of RCA [65,102]. However, there are still some common problems and challenges, including the following.

1. Consistency of Enhancement Effects: The physical and chemical properties of RCA may vary significantly due to differences in the source materials, leading to inconsistent enhancement effects reported in the literature. Therefore, it is necessary to conduct in-depth research into the changes in the properties of RCA and the mechanisms of enhancement to achieve more stable enhancement effects for practical engineering applications.
2. Compatibility of Composite Materials: The compatibility between RCA and enhancement materials (such as polymers, microorganisms, etc.) is a crucial issue. Researchers need to explore the interactions between suitable enhancement materials and RCA to improve the performance and stability of composite materials.
3. Long-Term Performance and Durability: While RCA enhancement techniques can improve mechanical properties in the short term, long-term performance and durability, especially at the component level, still need further investigation. Researchers should focus on the aging of materials, fatigue performance, and durability of RCA after enhancement to ensure their reliability in real-world engineering applications.
4. Feasibility for Large-Scale Applications: RCA enhancement techniques need to be economically feasible and scalable for large-scale applications. Therefore, researchers should consider the scalability, cost-effectiveness, and practical implementation of enhancement techniques to promote their widespread adoption in real engineering projects.

The future research directions of recycled concrete aggregate (RCA) enhancement may encompass the following areas:

1. In-Depth Study of Strengthening Mechanisms: Further in-depth studies of the mechanism of recycled aggregate strengthening technology, including the interaction between reinforced materials and recycled aggregate and the formation mechanism of the strengthening effect, should be performed in order to reveal its basic principles and influencing factors and provide a theoretical basis for surface modification of recycled aggregate and particle grading design optimization.
2. The Development of New Strengthening Materials: New strengthening materials with better compatibility, strengthening effects and environmental friendliness, such as nanomaterials, fibre materials, biomaterials, etc., should be explored to improve the technical performance and stability of recycled aggregate and reduce the production cost of recycled aggregate.
3. Integration of Multi-functional Strengthening Technology: Different recycled aggregate strengthening technologies are integrated to form a multi-functional strengthening system. For example, polymer strengthening, microbial induced carbonization deposition, and other technologies should be combined to achieve a more comprehensive recycled aggregate strengthening effect that is conducive to the promotion and application of recycled aggregate in high-strength concrete, ultra-high performance concrete, and other fields.
4. Sustainability and Environmental Impact Assessment: A comprehensive sustainability assessment of recycled aggregate strengthening technology should be performed considering its sustainability in terms of resource use, energy consumption, environmental impact, etc. There is also a need to study the potential environmental and human health impacts of recycled aggregate-strengthening technologies. Under the premise of not affecting the workability, mechanical properties, and durability of concrete, the highest replacement rate of recycled aggregate is studied to realize the recycling and efficient utilization of waste concrete and reduce the cost of recycled concrete.

In summary, future research efforts should be dedicated to addressing the challenges associated with RCA enhancement techniques and promoting their feasibility and sustainability in practical engineering applications. This will contribute to enhancing the performance of recycled concrete aggregates, advancing sustainable construction, and promoting the recycling of resources.

## Figures and Tables

**Table 1:** The CO_2_ emissions reductions from the use of carbonized solid waste in mortar and concrete in typical countries [66].

Region	Countries	Indirect CO_2_ Reduction (Mt)
North America	Canada	45.5
	United States	464.9
	Mexico	5.9
Latin America	Brazil	53.5
Europe	Germany	226.9
	United Kingdom	106.6
	Netherlands	28.8
	Belgium	16.2
	France	298.6
	Spain	41.6
	Turkey	14.9
	Russia	43.2
Africa	Guinea	20.3
	South Africa	17.9
Asia	China	1603.7
	India	175.2
	Japan	149.6
	South Korea	42.0
	Saudi Arabia	5.0
	Indonesia	18.8
Oceania	Australia	107.7
